# Systemic support for learners with developmental language disorders in Zimbabwe and South Africa

**DOI:** 10.4102/sajcd.v69i1.850

**Published:** 2022-02-16

**Authors:** Nettie N. Ndou, Margaret F. Omidire

**Affiliations:** 1 Department of Educational Psychology, Faculty of Education, University of Pretoria, Pretoria, South Africa

**Keywords:** developmental language disorder, inter-professional collaboration, inclusive education, systemic support, teaching and learning, medium of instruction, second language

## Abstract

**Background:**

Teachers play a significant role as early identifiers of learners with developmental language disorder (DLD). They provide important information to other professionals for further specialist support of such learners. Professionals, such as educational psychologists, speech–language therapists (SLTs) and learning support therapists are involved in assisting learners with DLD; hence, inter-professional collaboration (IPC) amongst these professionals is of paramount importance in meeting the needs of learners.

**Objectives:**

This study aimed to examine systemic support strategies available to learners with DLD.

**Method:**

This was a multiple case study of Zimbabwe and South Africa. Purposive sampling was used to select participants. The study consisted of 56 participants: 5 teachers, 2 SLTs, a learning support therapist, an educational psychologist and 47 learners. A qualitative research approach was employed and data were collected using interviews, focus group discussions and classroom observations. The data were analysed thematically and categorised.

**Results:**

Support strategies employed by teachers include remedial lessons and promoting a culture of reading for leisure to enhance learners’ vocabulary and narrative skills. The SLTs and the learning support therapist use speech–language programmes and assistive technologies. Limited IPC and the absence of SLTs in District Based Support Teams were some of the challenges identified. The results also indicate that SLTs receive referrals mostly from primary schools compared with secondary schools.

**Conclusion:**

Raising awareness of DLD in schools and communities is deemed essential. Inter-professional collaboration is recommended to support learners with DLD as it increases the exchange of ideas and mutual acknowledgement of expertise amongst professionals.

## Introduction

Developmental language disorder (DLD) is a condition in which there are unexplained persistent difficulties with language. Learners with DLD experience significant challenges in learning, understanding and using spoken or written language (McGregor, 2020). This is exacerbated when teaching and learning takes place in circumstances where the medium of instruction (MoI) is a second or additional language as is the situation in many schools in both Zimbabwe and South Africa. In these instances, the learners have further difficulty developing cognitive academic language proficiency (CALP). At secondary school, learners are required to use complex vocabulary and more complex sentence structures in a second language and for learners with DLD, which can be a challenge (Ossai & Uzoegwu, 2019). Inclusive education (IE) guidelines stipulate that such learners must be supported to thrive like typical developing learners. Bronfenbrenner’s (1990) ecological systems theory on human development suggests a learner’s ecology, which is made up of interwoven network systems to support learners with special needs such as DLD (Panopoulos & Drossinou-Korea, 2020). It has been suggested that inter-professional collaboration (IPC) amongst speech–language therapists (SLTs), educational psychologists, audiologists, social workers and teachers is likely to reflect the needs of a learner with DLD (Liu et al., 2018; as cited in McGregor, 2020). However, IPC remains scarce in practice (Lakkala et al. 2021). Thus, there has been a continued concern on the support of learners with special needs such as DLD in the mainstream schools (Dockrell, Rickets, Palikara, & Charman, 2019), and the current scholarship is limited on the support of such learners (Joffe, Rixon, & Hulme, 2019). Hence, the main thrust of this study was to explore systemic support strategies for secondary school learners with DLD and gravitate towards more inclusive opportunities for them to learn.

## Inclusive education

Inclusive education is based on commonality and IPC amongst professionals, parents and learners (Lakkala et al., 2021). It is a reform that supports diversity amongst all learners (UNESCO, 2001). UNESCO (2005, p. 13) defines IE as ‘a process of addressing and responding to the diversity of needs of all learners through increasing participation in learning … reducing exclusion within and from education’. It promotes a flexible curricular arrangement, where all learners’ needs are accommodated (Sithole & Mafa, 2015). Inclusive education is widely supported in international human rights law, international conventions ratified by members and most human rights instruments. Such instruments include the Universal Declaration of Human Rights (United Nations, 1948), the Convention on the Rights of the Child (United Nations, 1989) and the Convention on the Rights of Persons with Disabilities (Majoko, 2016).

### Inclusion policy in Zimbabwe

Zimbabwe subscribes to all the above-mentioned international human rights instruments. Several policies and legislations mandate IE (Majoko, 2013). These include the *Education Act of 1987* as revised in 2006, the *Constitution of Zimbabwe Amendment Number 20 of 2013, Section 75, the Disabled Persons Act of 1996*, and recommended practice circulars, including the secretary’s circular Number 2 of 2000 and the Directors’ circular Number 7 of 2005 (Majoko, 2018).

The Department of Schools Psychological Services (SPS) and Special Needs Education (SNE) of Zimbabwe provide various programmes and services to facilitate access, participation, acceptance and achievement of all children in quality regular education (Majoko, 2018). These programmes and services include needs-driven expansion of national provincial and district education officers and school heads and teachers (Munjanganja & Machawira, 2015).

### Inclusion policy in South Africa

South Africa also endorses international instruments that support IE. In South Africa’s constitution, an explicit section on the rights of people with disabilities is included. Education White Paper 6 (EWP6) (Department of Education, 2001, p. 10) outlines the government’s new policies for a single and undivided education system for all learners (Danohue & Bornman, 2014). According to Danohue and Bornman (2014), EWP 6 is designed to change the South African education system through the inclusion of all learners using a curriculum that is more flexible and suitable for the needs of all learners, developing district-based support teams and school-based support teams (SBSTs). These teams shall provide systemic support for teachers who need it to cope with diverse classes.

It is important to highlight that EWP6 (South African Government [SA GOV.], 2001) constituted certain support structures to support learners with special needs, such as DLD. These support structures include a support team at the school level, namely the SBST. The team comprises mainly teachers at the schools but may also involve health professionals from the community. The District Based Support Team (DBST) consists of a variety of support professionals (learning support teachers, health professionals and curriculum specialists) who provide support to all schools in the district area (Nel & Grosser, 2016).

EWP 6 (SA GOV., 2001) says that the critical function of the DBST is to support all learners, teachers and the system as a whole so that the full range of learning needs can be met. The aim is to assist teachers in schools in creating greater flexibility in their teaching methods, assessing learning, evaluating programmes, diagnosing their effectiveness, suggesting modifications and providing direct interventionist programmes to learners. The key functions of the SBST are to coordinate all learners, teachers, curriculum and institutional development support within the school and identify institutional needs.

## The diagnosis of developmental language disorder

In the *International Classification of Diseases* (*ICD-10*) and in the *Diagnosis Statistical Manual* (*DSM IV-R*), on the definition of ‘specific disorder of language acquisition’, a distinction is made between expressive and mixed expressive-receptive (MLD) types of language impairment (Assous et al. 2018). In the *DSM-5*, language disorders are included in the neuro-developmental disorders category and ‘the distinction between expressive and mixed types has been removed’ (Assous et al., 2018, p. 2). The *DSM-5* diagnosis is also based on the basis of age-appropriate non-verbal and exclusionary criteria. Recent research also suggests that learners with DLD have deficits in linguistics and non-linguistics aspects of the language disorder (Thomas, Schulz, & Ryder, 2019).

In this article, the term DLD is used as it was recommended by the Criteria and Terminology Applied to Language Impairments: Synthesising the Evidence (CATALISE) panel. In 2015–2016, an international group of experts in the area of Speech, Language and Communication Needs, the CATALISE panel, came together to agree on children’s diagnostic terminology on language problems (Bishop, 2017). They recommended that the term DLD be used to refer to neuro-developmental language deficit (McGregor, Goffman, Horne, Hogan, & Finestack, 2020). This condition emerges in early childhood and frequently persists into adulthood, and it can affect a person’s ability to learn effectively, establish relationships and seek gainful employment (Cronin, 2017; cited in Walker & Haddock, 2021). Learners with DLD can be supported in the mainstream school where specialist provisions and services are provided, in special schools and in support centres where learners are supported as individuals or groups (Dockrell et al., 2019). This article focuses on the support of such learners within the mainstream schools.

## The support of learners with developmental language disorder in the mainstream schools

It has also been widely accepted that delayed or lack of diagnosis and intervention for learners with DLD often leads to poor education outcomes, longer periods of unemployment, low self-esteem and a high risk for depressive and anxiety disorders (Thomas et al., 2019). Therefore, such learners must be identified so that professionals would collaboratively offer them support. However, Bishop, Snowling, Thompson, Greenhalgh and Catalise Consortium (2016) stated that the complex and multifaceted nature of language adds to the difficulties of identifying and categorising DLD. As a result, learners with DLD are not always easily recognised. Many learners with DLD may have an undetected or hidden difficulty with language acquisition. This is because such learners often develop compensatory strategies, for example, always agreeing or disagreeing with the conversational partner, remaining silent or responding using learned phrases (National Behaviour Support Service [NBSS], 2011; Walker & Haddock, 2021).

In an inclusive teaching and learning environment, pedagogy, curricula and assessment are planned and delivered to engage all learners in learning that is meaningful, relevant and accessible (Hewett, Douglas, McLinden, & Keil, 2020). Hamre and Pianta (2005) observed that schools need to set high expectations for what every child can achieve. These high expectations can be complemented with support structures and services: positive learning environments offer strong instructional and emotional support. Hence, teachers must be knowledgeable in the identification of learners who may need additional support (Prezas & Ahyea, 2017). Although teachers are not responsible for making decisions related to language proficiency or assessment, they play a very important role as early identifiers of learners who may or may not need further observation or assessment (Prezas & Ahyea, 2017). Consequently, identification and referral of learners with DLD to other professionals for further assistance is commonly the teachers’ responsibility. Responsibility is placed on teachers as they spend more time with the learners (Christopulos & Kean, 2020). They provide important information (e.g. academic or social) to other professionals for determinations of educational need for services in the schools.

However, limited understanding of DLD by teachers negatively affects learners with DLD in the mainstream schools, they remain undiagnosed. Teachers’ limited awareness may be explained by the fact that the signs of DLD are difficult to track (Adlof, 2020). After conducting a study, Christopulos and Kean (2020) concluded that mainstream teachers are responsible for most referrals made to special education but demonstrated difficulty in correctly identifying learners with DLD. Inaccuracy in identifying and referral process by teachers has been attributed to a lack of understanding of language structure and learners’ language needs (Christopulos & Kean, 2020).

Limited awareness of DLD is also a result of a cacophony of terms that have been used to explain DLD (Bishop, 2014). For instance, language delays, specific language deficit, specific language impairments and deviant language. Hence, limited awareness amongst the general population has resulted in inadequate service delivery for supporting learners with DLD and limited number of research studies in this area (McGregor et al., 2020).

The use of a second language (L2) as the MoI inevitably affects many learners and more so those learners with DLD (Tribushinina, Dubinkina-Elgart, & Rabkina, 2020). The presence of language difficulties has a significant impact on the academic success and educational attainment of learners with DLD. Often, such learners’ function at the basic interpersonal communication skills (BICs) level and do not develop CALP (Lillywhite, 2011). Academic language skills include the use of inferential language, that is, communicating about ideas across contexts, use of narrative language, where learners are expected to describe a series of events and understanding of the range of academic vocabulary and grammatical structures (Wissel, 2016). It is therefore not surprising that learners with DLD are in a dire situation in terms of poor academic achievement (Tuite, 2019). Still, there is limited literature on the effect of the use of L2 as MoI on learners with DLD (Tribushinina et al., 2020; Zoutenbier & Zwitserlood, 2019). It is against this background that there is need to support such learners, and because of the complex and multifaceted nature of DLD, IPC is necessary to effectively identify and support them.

### The role played by inter-professional collaboration in support of learners with developmental language disorder

Teachers offer initial support to learners with DLD to enhance learners’ language skills in the areas of vocabulary, narrative and expository skills. The complexity of language use increases in secondary schools and teachers must break down large amounts of information into shorter paragraphs (Roehling, Herbet, Nelson, & Boharty, 2017). Teachers also play a primary role in the identification of learners with special needs under a referral-based format (Christopulos & Kean, 2020). Usually, professionals such as SLTs, educational psychologists and learning support therapists’ work begins when teachers have challenges with assisting learners with DLD in the classrooms.

Inter-professional collaboration has been recommended as a means by which the needs of learners with additional needs can be met (Gallagher et al., 2020). Inter-professional collaboration is a shared expertise, whereby team professionals create strategies together, learning from professionals in other fields (Lakkala et al., 2021). Speech–language therapists contribute specialist knowledge and skills regarding learners with DLD; they are specialists trained to diagnose and assist learners in speech–language and communication needs (SLCNs). Hence, SLTs have an integral role in education. In linguistics aspects, they assess grammar, phonology and semantics, whereas in non-linguistics aspects, they assess phonology awareness, phonological short term and working memory. These are addressed in tests such as Clinical Evaluation of Language Fundamentals (CELF-5) and the Comprehensive Test of Phonological Processing 2 (Thomas et al., 2019).

Augmentative and alternative communication (AAC) devices are also used to support learners with DLD (Uthoff et al., 2021). Augmentative and alternative communication tools and strategies include visual schedules, picture and written supports for spoken language. According to Speech-Language and Audiology Canada (SAC) (2015), AAC tools and strategies for supporting expressive communication include object or picture-based choice making, communication displays, alphabet boards, symbol and text-based speech-generating devices and alternative access methods.

Intervention models have been proposed for the effective support of learners with DLD. Prezas and Ahyea (2017) proposed a multi-tiered support system for learners with DLD. This tiered framework is used to provide early detection and prevention through academic and or behavioural support to learners. A response to treatment model of intervention has also been proposed to identify learners at risk of poor learning outcomes and to identify learners with speech, language and communication needs (Walker & Haddock, 2021). Ebbels, McCartney, Slonims, Dockrell and Norbury (2019) also presented a tiered intervention model for learners with DLD. These models recommend a strong IPC amongst professionals in assisting learners with DLD.

Whilst the first two models emphasise the role played by SLT in assisting learners with DLD at the last stage or tier, Ebbels et al. (2019) emphasised that the role of SLTs must not only begin at the last stage or tier but they should also offer the education field guidance and support throughout the process of educating. They must influence public awareness and policies, provide advice and problem-solve evidence-based programmes. They must also be active in assessment, planning, training and monitoring others delivering indirect intervention and monitoring of progress, as well as being actively involved in assessment and monitoring of progress on an individual learner (Ebbels et al., 2019).

However, IPC is a complex phenomenon and a collaborative advantage is difficult to achieve (Gallagher et al., 2019). This leaves many learners at risk of poor social, emotional and educational outcomes (Gallagher et al., 2020). Professionals involved in assisting learners with DLD come from different backgrounds, which make it difficult to share a common understanding of DLD (Gallagher et al., 2020). Although much had been done in the past years to address terminology and criteria to identify learners with DLD (Bishop, 2017; Bishop et al., 2016), there is still not sufficient consensus amongst professionals who support learners with DLD.

A review of the literature on IPC between teachers and SLTs in the support of learners with DLD concluded that there was little common understanding of DLD between the professions. These different views are also evident in practice. The literature concurs that although much had been done in the past five years to address issues of terminology and criteria to identify learners with language disorders (Bishop, 2017; Bishop et al., 2016), there was still not sufficient consensus between these professions. Current scholarship lacks crucial insights into collaborative work between teachers and SLTs on intervention strategies to support learners with DLD. Based on the literature reviewed, most literature on DLD originates in the speech–language therapy field of inquiry rather than the education field of inquiry.

Furthermore, the referral-based system works well when teachers have the expertise of identifying learners with DLD. However, in the context of limited training and knowledge about identification and support of learners with DLD by teachers, very few learners are being supported (Roberts & Simpsons, 2016). The knowledge of typical language development in children is essential to recognise indicators that a learner may be presenting with signs of DLD. Information about speech–language development is however often minimal or absent in many initial teacher training programmes (Walker & Haddock, 2021).

A lack of awareness of DLD by teachers may also be explained by the fact that the signs of DLD are difficult to track (Adlof, 2020). Furthermore, proper identification of DLD by teachers is made even more complicated in a multilingual context (Belanger, Mayer-Crittenden, & Minor-Corriveau, 2020) as DLDs are often confused with language barriers. It must be observed that the terms language ‘disorder’ and language ‘barrier’ is different and cannot be used interchangeably. Therefore, limited awareness of DLD amongst teachers greatly affects effective identification and support of learners with DLD.

The scarcity of trained professionals such as SLT to work with learners with DLD and limited evidence of base underpinning effective interventions have raised serious concerns on the support of learners with DLD (Dockrell et al., 2019), yet there has been no attempt to map the support of such learners in the mainstream schools (Dockrell et al., 2019). It has been observed that there are limited SLT services and specialist provisions particularly for secondary school learners with DLD (Dockrell et al., 2019).

Limited support of such learners in the mainstream secondary settings negatively affects learners with DLD. Harthshorne (2011) explained the challenges faced by learners with DLD in secondary schools as a ‘cycle of neglect’. Many of these challenges are interrelated and impact each other. When very few learners are identified with DLD in secondary schools, there are restricted services for learners with DLD that do not reflect the actual level of needs. There is limited awareness of the importance of speech–language communication needs by teachers, general school staff and the community as there is an assumption that language development and early intervention only happens early in the primary years and not in secondary school. There is a narrow understanding of the role of language in the secondary school curriculum. With time, there are fewer professionals with skills and knowledge to support learners with DLD and limited resources and assessment tools for early identification and support of such learners.

In Zimbabwe, there are limited support services for learners with DLD. Cooley (2007) observed that ideally learners with DLD should be assisted by SLTs in schools. However, it is important to observe that this may not be feasible in developing countries such as Zimbabwe. Zimbabwe desperately needs SLTs to work in public institutions such as schools. Currently, there are very few SLTs working in public service in Zimbabwe. This was also confirmed by the permanent secretary in the Ministry of Health and Child Care, Dr. Gerald Gwinji, who argued that the government does not have money to pay specialists’ salaries. He added that Zimbabwe trains SLTs but cannot retain them because their expertise is highly marketable and they work for private organisations (Hutchins, 2016).

In addition, lack of support from the SNE is another challenge facing teachers in schools in the support of learners with special needs such as DLD. Inclusion is grounded in the social, physical, cultural and emotional integration of learners with unique needs and those who are at risk of exclusion and marginalisation in general education (Majoko, 2018). Ideally, the SPS and SNE place and support learners with special needs in mainstream classrooms (Majoko, 2018); however, limited involvement of these specialists has led to overburdening of teachers. Hence, the functions of SPS and SNE have become the responsibility of teachers who also do not have expertise in assisting learners in need (Sunal & Mutua, 2013).

In South Africa, learners with DLD fail to achieve the necessary outcomes in language and literacy. However, SLTs do not play an effective role in public sector education because of several factors; notably, the current human resource capacity of SLTs is severely limited (Kathard, Jordaan, Wium, Pottas, & Khan, 2011). The available human resource data indicated that there are ‘186 speech therapists across the country appointed at special schools, whilst there were 73 ‘office-based 46 therapists (a general category that includes occupational therapists and physiotherapists)’ (Kathard et al., 2011, p. 65). Furthermore, Southwood and Dulmo (2015) observed that there is still an absence of appropriate assessment and remedial material for Afrikaans and African languages (Southwood & Dulmo, 2014).

### Linking Bronfenbrenner’s (1990) ecological systems theory in the support of learners with developmental language disorder

The conceptual framework utilised in this study was Bronfenbrenner’s (1990) ecological systems theory on human development in explaining IE’s ecology. Inclusive education is based on a belief that the inclusion of children with special needs in mainstream schools is beneficial for their studies and growth (Nai-Kwai Lo, 2007). Bronfenbrenner’s theory’s central point relates to the interactions created between the developing and active individuals and the social context that surrounds them and influences them directly and indirectly (Panopoulos & Drossinou-Korea, 2020). The theory presents five ecological system theories that influence the psychological development of a learner. These are the microsystem, mesosystem, exosystem, macrosystem and chronosystem (Bronfenbrenner, 1990). These five systems describe the interwoven networks of transactions that create a learner’s ecology.

Teachers must identify and support learners with DLD early in their school career. Teaching methods used by teachers must be inclusive to meet the needs of learners with DLD. Teachers must be equipped with the necessary skills to assist learners with DLD in the mainstream classroom (microsystem). Inter-professional collaboration is essential to meet the needs of the affected learners. Hence, professionals such as SLTs, learning support therapists and educational psychologists must work closely with teachers in supporting the learners (mesosystem). Other support structures such as the family are also important in meeting the needs of affected learners.

The general school environment must be inclusive to cater to the needs of all learners. The school timetable must accommodate support services offered by teachers or other professionals (exosystem). Educational policies such as IE must be implemented to meet the needs of learners (macrosystem). Finally, learners with DLD receiving support at the primary level of education must continue receiving the necessary support at the secondary level of education (chronosystem).

## Purpose of the study

The study sought to examine systemic support strategies in the inclusion and support of secondary school learners with DLD. Although there is evidence of effective support strategies for learners with DLD in pre- and primary school-aged learners, there is limited evidence of effective strategies to support secondary school learners with DLD (Dockrell et al., 2019; Joffe et al., 2019). Based on the purpose of the study, the research question guiding the study was what are the current support services available for secondary school learners with DLD?

To answer this research question, a qualitative approach was applied to find out strategies used by professionals to support secondary school learners with DLD.

## Research methods and design

The study was designed as a qualitative study within the interpretive paradigm employing a multiple case study design. A case study investigates a real-life case or cases over time through detailed, in-depth data collection involving multiple sources of information (Creswell, 2013). The two cases selected were situated in Zimbabwe (Case 1) and South Africa (Case 2). A multiple case study enabled an investigation of systemic strategies used to support learners with DLD in two different contexts. This design allows investigation of the phenomena under study through the use of a replication strategy. This made this study more interesting as the data analysed were derived from two different contexts (Diop & Liu, 2020).

### Setting

The study was carried out in five secondary schools: two located in Bulawayo Khami district in Zimbabwe and three located in Johannesburg Central district in South Africa. The secondary education system in Zimbabwe lasts for six years. It consists of lower secondary and upper secondary. The lower secondary level is a four-year cycle: from 1 to 4 that leads to awarding of the ordinary level certificate. At upper secondary, learners are offered advanced level studies meant to prepare them for university education (The Republic of Zimbabwe, 2019).

Similarly, secondary education in South Africa lasts for six years in duration (from grade 7 to grade 12). It is also divided into lower (senior phase) and upper secondary further education and training (FET). The senior phase lasts through grade 9 and FET lasts through grade 12 (Macha & Kadakia, 2017).

### Study participants and sampling strategy

The inclusion criterion was based on the aim to gather evidence from experienced professionals who work with learners with DLD showcasing their first-hand knowledge and supporting such learners. Hence, participants were purposively selected. In Zimbabwe, three schools were purposively selected to take part in the study. This decision was based on familiarity with the location. However, one school withdrew from the study. In school A, 10 learners and 1 teacher were part of the study, and in School B, 15 learners and 1 teacher were part of the study. Other professionals, such as the SLT and the learning support therapist, also participated in the study.

In South Africa, three schools were purposively selected and the decision was also based on geographical location and familiarity with the location. In school C, nine learners and one teacher were part of the study. In school D, eight learners and one teacher participated in the study, and finally, in School E, five learners and one teacher were part of the study. The SLT and an educational psychologist also participated in the study.

From both countries, learners who participated in the study were in the lower secondary level of education. Their age ranges between 13 and 17 years. Teachers were English language teachers. Teachers were requested to select learners with DLD in their respective schools; this selection was based on their understanding of DLD. The number and nature of participants were determined by the nature of the study; being a qualitative study, the desire was to explore in-depth systemic strategies used to support secondary school learners with DLD.

### Data collection

A set of the semi-structured interview guide was developed based on the topic under study. Each question was intended to stimulate discussion on issues relevant to the research questions. Interviews were conducted with nine professionals. Classroom observations and focus group discussions were also used as means of collecting data for research. Table 1 presents a summary of the data generation process in Zimbabwe and South Africa, respectively.

**TABLE 1:** Data generation process–summary structure for Zimbabwe and South Africa.

Date 2019	Maximum duration	Activity	Participants
21–03	30 min	Introductory meeting with the Provincial Education Director’s secretary	-
1–04	15 min	Introductory meeting with the District Inspector	-
28–02	30 min	Introductory meeting with the principal from school B	-
21-03	30 min	Introductory meeting with the principal from school A	-
4-04	1 h	Choosing participants and signing contracts	Learners in School A (10)
4-04	1 h	Choosing participants and signing contracts	Learners in school B (15)
8-04	1 h	Focus group discussions completing demographic questionnaire	Learners in school A
8-04	30 min	Semi-structured interview (School A)	Teacher A
		Semi-structured interview (School B)	Teacher B
12-04	1 h 30 min	Semi-structured interview	Learning support therapist
16-05	1 h	Semi-structured interview	SLT
13-06	35 min	Classroom observation	School A
12-07	35 min	Classroom observation	School B
12-07	30 min	Focus group discussion	School B
15-04	1 h	Introductory meeting with the Gauteng province, central district	-
15-04	1 h	Introductory meeting with the DBST	-
12-08	30 min	Introductory meeting with the principal, choosing participants and signing contracts	School D
12-08	30 min	Introductory meeting	SLT
13-08	1h 30 min	Semi-structured interview	SLT
13-08	30 min	Introductory meeting with the principal, choosing participants and signing contracts	School E
14-08	1 h	Interview	Teacher E
14-08	30 min 1 h	Focus group discussions	School E
		Semi-structured interviews	Teacher E
15-08	1 h 30 min	Classroom observation	School C learners
		Semi-structured interview	Teacher C
16-08	30 min 1 h	Focus group discussions	School D learners
		Semi-structured interviews	Teacher D
19-08	-	Classroom observation	School D
20-08	1 h	Semi-structured interview	Educational psychologist

SLT, speech–language therapist; DBST, District Based Support Team.

### Data analysis

Inductive thematic analysis was used to analyse the data. Thematic analysis is a method for analysing qualitative data involving searching across data set to identify, describe and interpret data, analyse and report repeated patterns (Kiger & Varpio, 2020). Coding helped to reduce the data into themes that emerged from the analysed data (Plano & Creswell, 2010). Atlas ti was used as data analysis program software. Transcriptions were checked for accuracy, and the files were entered into Atlas ti for analysis.

### Trustworthiness

To ensure the trustworthiness of the research findings, engagement with participants was prolonged and data sources (semi-structured interviews, focus group discussions and classroom observations) were triangulated. Space triangulation (Case 1 in Zimbabwe and Case 2 in South Africa) was also used in an attempt to overcome parochialism (Cohen, Manion, & Morrison, 2011). Another reliability test to ensure trustworthiness was member checking, where transcribed data were emailed to adult participants for verification (Gunawan, 2015).

### Ethical considerations

Ethical clearance was obtained from the Faculty of Education, University of Pretoria (Clearance number: EP 18/10/01). Permission to conduct research in Bulawayo Khami district in Zimbabwe and Johannesburg central district in South Africa was granted. Informed consent, confidentiality and voluntary participation were incorporated throughout the study.

## Results

Three themes emerged from data analysis, conceptualisation of DLD, support services available for learners with DLD and the effect of using a second language (L2) as the MoI on learners with DLD. However, this article focuses on the second theme: support services available for secondary school learners with DLD. This theme yielded four sub-themes. The qualitative study provided a credible and rich description of the systemic strategies used by professionals in supporting such learners.

### Teachers

**FIGURE 1:**
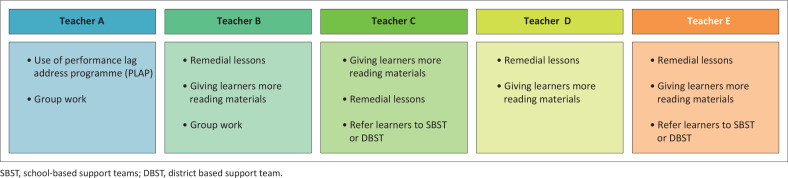
Services offered by teachers.

Teachers explained how they identify learners with DLD in the classroom. According to Teachers A and B, learners with DLD struggle to read and understand a comprehension passage and fail to narrate a story’s events. Such learners cannot construct proper sentences and make spelling challenges. Teacher C mentioned that learners with DLD tend to make spelling errors. They also fail to copy correctly what is written on the board. Teacher E argued that learners with DLD fail to construct proper sentences and cannot spell words correctly. She added that during reading sessions, such learners do not come for the lessons. If they are in the classroom, they try by all means not to be picked out to read, have some stammer and lack confidence. Figure 1 summarises support services offered by teachers to support learners with DLD.

Teacher A used the Performance Lag Address Programme (PLAP) to help learners with DLD. The programme assists learners to learn simple English and mathematical skills. For a learner with DLD, PLAP helps learners to read stories and discover some new words. Teacher A also uses group work; she argued that in groups, learners help one another discover new English words. Teacher B conducts remedial lessons to assist learners with DLD. She groups the learners and helps them to learn some new words and concepts at a slow pace. She sometimes gives them beginners’ readers such as the Sunrise collection and they learn so many new simple English words. She also uses group work to assist the learners. She argues that learners in groups complement each other and they will be assisting each other.

Learners confirmed support strategies used by their teachers. They confirmed that they are given books to read on their own. Sometimes they are put in groups and do some tasks as a team. One learner also mentioned that their teacher once grouped several learners who had challenges in understanding some concepts and assisted them. However, the use of the terms ‘sometimes’ and ‘once’ by learners explains the support strategies’ frequency. It can be deduced that teachers do not regularly assist the learners.

Teachers explained that they do their best to assist learners with DLD, although they are not qualified specialists in special needs. Learners with DLD also receive limited support due to insufficient contact hours with the teachers. Teacher–learner ratio and minimum parental involvement were also highlighted as challenges that hinder effective implementation of the support strategies. Furthermore, teachers also identified a need for more support from the SPS and SNE Department in assisting learners with special needs.

In South Africa, Teacher C explained that learners with DLD are called ‘learners at risk’ in the school. He argued that they are at risk because there is a high probability of their failure in their Grade 12 exit examination. He argued that they design an intervention plan or a remedial plan to assist the learner. Teacher C also mentioned that in some cases, where a learner presents with a severe DLD, they refer the learner to the DBST for further assessments and assistance. Teacher D highlighted that she gives learners with DLD more reading books and sometimes conducts remedial lessons. However, she stressed that she could not offer them long-term services. She argued that she receives little support from the DBST. She mentioned that:

‘[*T*]he problem is there is no one to help these learners, the system depends on me trying different ways to assist, one thing they encourage is that they call it differentiation … but you realise I don’t have the time, so we normally do what I call ‘quick fix …’ (P33, SI, ln20–25)

She added that:

‘I can’t offer a long-term service. It’s just what can I do to make you pass and proceed to the next grade, so it’s not something long term … there are no support structures …’ (P33, SI, ln35–38)

Furthermore, Teachers C and D argued that the team of SBST is made up of unqualified special education teachers who are full-time teachers with a full workload. Hence, it becomes challenging to assist learners with special needs such as DLD. Teacher C highlighted that:

‘The SBST assists such learners, but the problem is they are not qualified special education teachers, they are just qualified in their subject area … they are also full-time teachers with their workloads, so they are overwhelmed.’ (P32, SI, ln27–30)

Teacher E also added that they are required to complete an accommodation form for a learner with special needs to be submitted to the DBST; however, most teachers evade completing the form because it is too long. Teachers also argued that they do not have the expertise to assist learners with DLD; hence, it becomes a challenge to identify such learners fully for further assistance. Other challenges highlighted by teachers were class size, limited contact hours, lack of cooperation from the learners and limited parental support, absence of a SLT in the SBST and DBST, limited expertise in assisting learners with DLD.

### Speech–language therapists

In Zimbabwe, the SLT highlighted the following services they use to support learners with DLD: screening, development of individual education plans (IEPs), development of speech programmes and the use of speech training mirrors. He explained that they have a checklist they use to screen learners. In screening, they gather information from parents and teachers regarding the learners’ language skills, conduct the hearing screening, administer some tests or even use observation methods.

The SLT argued that they first train teachers on how to identify learners with DLD and how to implement the speech training programmes. The SLT clarified the services they offer secondary school learners with DLD. His accounts are shown here:

‘[*I*]n principle, we do have programmes for learners at the secondary school level, but when we go there, we find out that the majority of our secondary school learners do not want to be identified with such programmes. So, I wouldn’t say we have the functional language speech programmes for them as such, but in primary schools, yes, we do.’ (P01, SI, ln24–28)

He added that:

‘[*L*]earners in the secondary school … are not willing to take up these programmes, but the programmes are there. I think there is some stigma that is attached to one being found in this speech programme when you are in Form 4 or 3.’ (P01, SI, ln108–110)

The SLT was also requested to clarify whether secondary school teachers were aware of support services on offer to support such learners with DLD. He explained that very few secondary school teachers were aware of DLD. He argued that most people are aware of physical disabilities as they can be seen, but when it comes to neurodevelopmental disorders such as DLD, most teachers are not aware. He explained that very few secondary school teachers are willing to take up duties in the SNE Department. Here is what he said:

‘[*W*]e need to train teachers; they come out of college without any background on learning disability … it is the duty of the teachers to assist these learners to be identified so that we can assist them … so the problem is in our higher learning institutions.’ (P01, SI, ln128–139)

He also showed his concern for secondary school learners with DLD who do not want to be associated with SNE. These were his views:

‘[*T*]here are many learners with language problems at primary level but very few at a secondary level … would it be that they did not proceed to secondary school than [*we*] will say no, the majority do proceed … maybe there are there in secondary school … they are afraid of their peers.’ (P01, SI, ln122–127)

The SLT highlighted that there are programmes designed for learners with DLD in secondary schools but they are not ‘functional’. Therefore, SLTs concentrated more on supporting primary school learners with DLD. This was also confirmed by one learner who said:

‘I have never seen other people coming here to help us, but some came to my school whilst I was in Grade 5 at [*primary school*]. They would come to teach us, give us some words and then we were tasked to pronounce … they only came two times …’ (L08, FGD, secondary school learner)

In South Africa, the SLT mentioned that they first give learners some tests as a screening process, and if a learner fails, then they do an in-depth assessment. The SLT highlighted that they then do screening for speech–language ability. At this stage, they identify learners with DLD. She added that they have speech programmes that they use to assist the learners with language stimulation services, where they introduce vocabulary to a learner to enrich a learner’s vocabulary. They also have AAC devices. These are divided into two categories: non-electronic AAC includes pictures, symbols, spellings and phrases to communicate, whilst electronic AAC includes the use of any electronic equipment to communicate, such as microphones and voice amplifiers. The SLT also mentioned that they mostly receive learners from the primary level of education. She also argued that they are under pressure because they are only two of them serving learners from both primary and secondary schools in the district.

### Learning support therapist

**FIGURE 2:**
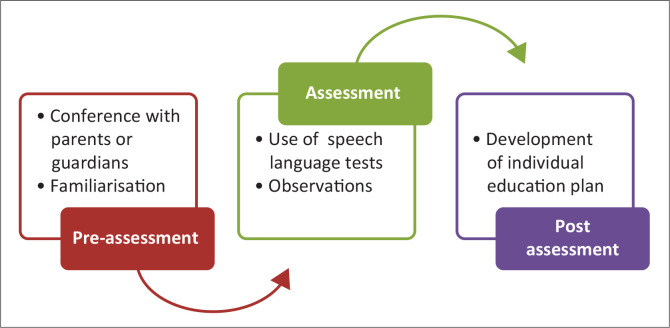
Assessment phases followed by the learning support therapist.

The learning support therapist explained that they conduct their diagnosis in three phases. Figure 2 presents the assessment phases used by the learning support therapist to identify learners with DLD.

In the pre-assessment phase, parents or guardians of the learner are asked questions about the family’s history and background. He explained the pre-assessment as follows:

‘[*W*]ill be asking questions about history in the family background, remember learning difficulties are genetic; were these milestones achieved on time or delayed? … we then bring the child for familiarisation.’ (P02, SI, ln21–26)

The second phase is the assessment phase. The learning support therapist highlighted that they determine the nature of the challenge facing the learner. For example, for a learner with DLD, the SLT run a series of speech–language tests with the help of another specialist. A full report on the learner’s condition is presented to the parents or guardians. In the final phase, they develop an IEP for the learner and they involve the learner’s school class teacher. They also do a pull-out system where the learner will be attending sessions three or four times a week for an hour or two. However, this depends on the parents’ or guardians’ ability to pay as most of their services are quite expensive. They also have assistive technologies such as C-Pen Readers and the KURZWEIL 3000 app, which help learners with DLD. However, these assistive technologies are expensive to buy and maintain.

### Educational psychologist

An educational psychologist highlighted that she first conducts a full cognitive assessment of the learner. She commented:

‘I do the full cognitive assessment … So, once I get the intellectual image of a child; IQ of the child, if it is average or above my expectations, even just a little below average … am expecting this child to be able to read and write, even if it … But you find some students have high IQ, the above average, some functioning super but they still can’t read and write then the learner has difficulties in language acquisition.’ (P31, SI, ln6–13)

She added that she does not offer support services, but she recommends the learner to the SLTs or audiologists after her series of tests.

## Discussion

Five systems outlined by Bronfenbrenner’s theory describe the interwoven networks of transactions that create a learner’s ecology. The school is the most important microsystem of a learner. The school environment, the learning process and the relationships learners with DLD develop with their peers and teachers, contribute to their social, cognitive and academic development (Anderson, Boyle & Deppeler 2014). Therefore, elements within the school (the curriculum, teaching methods, learners, teachers, the principal, support teams and their relationships) must support learners with DLD to realise their potential. Hence, strategies to support learners with DLD are required.

The research findings indicated that teachers use several strategies to support learners with DLD. These strategies enhance learners’ vocabulary and narrative skills and have been proven by several scholars as effective (Lowe & Joffe, 2017; Murphy et al., 2017; Spencer et al., 2017). However, teachers highlighted challenges that negatively affect the smooth implementation of these strategies: lack of expertise in assisting learners with DLD, limited contact hours with the learners and the class sizes, which do not promote effective learner-centred learning.

It must be emphasised that more learners with special needs are being educated in mainstream classrooms than ever before, resulting in higher expectations of mainstream teachers’ abilities to meet the needs of such learners (Gilmour & Wehby, 2020). However, educational training for teachers in mainstream classrooms rarely prepares them for working in diverse classrooms. It does not equip teachers with the knowledge and skills to effectively support learners with special needs such as DLD (Zwane & Malale, 2018). This lack of skills limits teachers’ expertise and confidence in assisting learners with special needs such as learners with DLD. Teachers play a primary role in the identification of learners with special needs for referrals under a referral-based format (Christopulos & Kean, 2020), hence they must be equipped with skills and expertise in SNE for easy and early identification of learners who need extra support.

The study’s findings also indicate limited IPC amongst professionals who deal with learners with DLD. For instance, teachers identified a need for more support from specialists in assisting learners with special needs such as DLD. In contrast, the SLTs argued that they received very few secondary school learners for support. This shows a limited inflow of information between teachers and SLTs, who are the key specialists in assisting learners with DLD. This becomes a serious setback in support of secondary school learners with DLD. For many years, IPC has been recommended as a means by which the needs of children with additional needs can be met (Gallagher et al., 2019). Even the conceptual framework has shown that there is a need for the collaboration of professionals at the mesosystem level in assisting learners with DLD. However, the study results have shown that effective IPC remains rare in practice leaving many learners at risk of social, emotional and educational outcomes (Gallagher et al., 2020). Consequently, the functions of the specialists become the responsibilities of teachers who also do not have the expertise in the areas (Sunal & Mutua, 2013).

Class size can also affect teaching methods used by teachers. Over-crowdedness in the classroom creates negative attitudes amongst teachers towards learners with disabilities in inclusive settings (Chimhenga, 2016). This hinders the effective implementation of strategies to support learners with special needs such as DLD in mainstream secondary schools. Teachers cannot practice various methods such as higher-order questioning and active learning approaches; they are effectively confined to the chalk and talk instructional method (Marais, 2016; Ngwenya, 2019).

The SLTs highlighted that they had several speech–language programmes for learners with DLD. However, they argued that they received more primary school learners than secondary school learners with DLD. This means that many secondary school learners with DLD are not receiving support from the SLTs who are the specialists in SLCN. Whilst secondary school teachers show discontentment over minimal support from other professionals such as the SLTs, the SLTs argue that they receive referrals mostly from primary schools. For instance, the SLT from Zimbabwe highlighted that there are support services for secondary school learners with DLD, but they are not ‘functional’. This raises serious concerns about the support of such learners in mainstream secondary schools. So, limited involvement of these professionals has left teachers with the responsibility. Yet, teachers also have limited expertise in assisting such learners (Sunal & Mutua, 2013). There is evidence of lack of collaboration between secondary school teachers and SLTs (Gallagher et al., 2019). There is also limited inflow of information amongst the SPS, SNE Department and secondary school teachers.

## Significance of the study

The study’s significance lies in the fact that it fills a gap in literature and practice on the support of secondary school learners with DLD. The absence of in-depth studies on strategies to support secondary school learners with DLD constitutes a vacuum in the current understandings of strategies to support learners with DLD. As Joffe et al. (2019) pointed out that there is limited evidence of effective strategies to support learners with DLD in secondary schools. Yet to date, there have been limited attempts to provide learners with DLD professional support in the mainstream schools (Dockrell et al., 2019). Furthermore, there is still limited literature on the effect of the use of a second language as MoI on learners with DLD (Tribushinina et al., 2020; Zoutenbier & Zwitserlood, 2019).

## Limitations of the study

This study was a multiple case study carried out in two schools in Khami district and three schools in the Johannesburg central district. Whilst the sample size facilitated the in-depth exploration of the phenomenon under study, the results of the study were limited to the participants’ experiences, geographical location and nature of the schools. Strategies highlighted by the participants to support learners with DLD in the area of study may be different from what other schools do to support learners with DLD. As a result, research’s findings may not be generalised to the greater populations of the two countries.

## Recommendations

**FIGURE 3:**
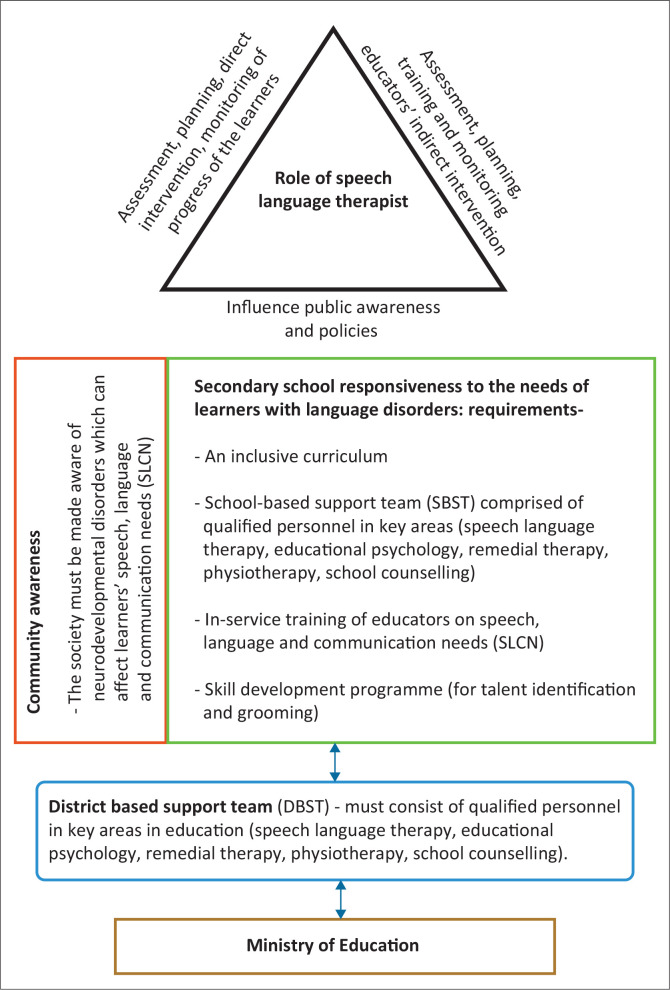
Proposed framework for supporting secondary school learners with developmental language disorder.

The framework adopts the ecology of an IE framework for systemic strategies to support learners with DLD (see Figure 3). A well-rounded curriculum and inclusive language of instruction that caters to all learners is recommended. In classroom instruction, teachers must apply both the ‘acquisition theory’ and ‘learning theory’ to language development. This way, all learners (typically developing and those with DLD) are accommodated. A school-based support structure that comprises qualified personnel in key areas in the education system (SLT, an educational psychologist, learning support therapist, counsellor and physiotherapist); in-service training of all teachers on SNE, for instance, in-service training can be offered on speech, language and communication needs and enlightening the community about common problems, which can affect children as they learn such as DLD. The role played by SLTs in assisting learners with DLD cannot be understated. Speech–language therapists’ duties in the school are to influence public awareness and policies, assessment, planning, direct intervention, monitoring of the progress of the learners and assessment, planning, training and monitoring teachers’ indirect intervention (Ebbels et al., 2019).

## Conclusion

Language in education plays a key role in effective teaching and learning worldwide. Yet, there is limited evidence of effective strategies to support learners with DLD in secondary schools (Joffe et al., 2019). This may be attributed to a limited understanding of DLD by teachers and may lead to inaccuracy in identifying learners with DLD (Christopulos & Kean, 2020). Limited awareness of DLD in local communities may also lead to very few learners with DLD being referred to specialists such as SLTs.

To achieve a systemic change in support of learners with DLD in secondary schools, there is a need for in-service secondary school teachers’ training on SLCN. If secondary school teachers are imparted with knowledge on SLCNs, more learners with DLD will be assisted and are accurately referred to specialists for further diagnosis and assistance. Zwane and Malale (2018) pointed out that education training for teachers in mainstream classrooms rarely prepares teachers for working in diverse classrooms. Therefore, the inclusion of in-depth SNE courses in the general teacher training courses is recommended. Teachers in-training must be fully prepared to work in inclusive, diverse classrooms.

Teachers also identified a need for more support from specialists in assisting learners with special needs such as DLD. Inter-professional collaboration between teachers and other professionals is the key to meeting the needs of learners with DLD. There must be smooth communication and cooperation between teachers and other professionals such as SLTs for effective support of learners with DLD. Hence, the implication is that there is a need for early identification, support and improved assessment tools with greater public awareness of DLD for secondary school learners with DLD to be fully supported.
